# Lycopene in Combination With Sorafenib Additively Inhibits Tumor Metastasis in Mice Xenografted With Lewis Lung Carcinoma Cells

**DOI:** 10.3389/fnut.2022.886988

**Published:** 2022-05-27

**Authors:** Ya-Ping Chan, Cheng-Hung Chuang, Inn Lee, Nae-Cherng Yang

**Affiliations:** ^1^Department of Food Science and Biotechnology, National Chung Hsing University, Taichung, Taiwan; ^2^Department of Nutrition, Hungkuang University, Taichung, Taiwan; ^3^Department of Nutrition, Chung Shan Medical University, Taichung, Taiwan; ^4^Department of Nutrition, Chung Shan Medical University Hospital, Taichung, Taiwan

**Keywords:** lycopene, anti-metastasis, adjuvant, sorafenib, MAPK pathway, NOX4

## Abstract

Cancer metastasis is the leading cause of death in cancer patients. However, it is unclear whether lycopene can act as an adjuvant to increase the anti-metastatic activity of anticancer drugs. Here, we examined the anti-lung-metastatic effects and the mechanism of lycopene in combination with sorafenib in C57BL/6 mice xenografted with Lewis lung carcinoma (LLC) cells. The mice were divided into five groups: (1) tumor control; (2) lycopene (5 mg/kg); (3) sorafenib (30 mg/kg); (4) lycopene (2 mg/kg) + sorafenib (30 mg/kg); (5) lycopene (5 mg/kg) + sorafenib (30 mg/kg). The results showed that lycopene reduced the number of metastatic tumors in the lungs, which was further suppressed by the combined treatment with sorafenib. The activities of matrix metalloproteinase (MMP)-2 and−9 were further inhibited and TIMP-1 and−2, and NM23-H1, the MMPs negative modulators, were further activated in the combined treatment. Mechanistically, we found that lycopene and sorafenib could additively inhibit the mitogen-activated protein kinase (MAPK) pathways, as shown by the protein phosphorylation of ERK1/2, JNK1/2 and p38 were reduced additively. Overall, the present study demonstrates that lycopene in combination with sorafenib additively inhibits the lung metastasis of tumor, indicating lycopene has potential as an adjuvant for sorafenib in cancer treatment.

## Introduction

According to the World Health Organization, lung cancer has the highest mortality rate of all cancers. In the United States, statistics from 2013 to 2018 show that men account for approximately 46.9% of lung cancer and women account for 32.0% (1). Cancer metastasis is a complicated process, which is one of the main causes of the recurrence and death of cancer patients. During the metastasis, cancer cells *in situ* move to other tissues and organs from the blood circulation or the lymphatic system through proliferation, adhesion, invasion, and migration. The metastasizing cells will proliferate in large numbers *in situ* and secrete proteolytic enzymes, such as the matrix metalloproteinases (MMPs), which degrade the extracellular matrix and blood vessel wall. The metastasizing cells penetrate the blood vessel wall by invasiveness, and migrate in the circulation, extravasate out of the blood vessels, colonize in the distant tissue, initiate angiogenesis, and finally grow at the new site (2, 3). Therefore, inhibiting cancer metastasis can be one of the strategies to effectively treat lung cancer.

Lycopene is a lipophilic natural compound with an acyclic and tetraterpene hydrocarbon compound containing 11 conjugated and two non-conjugated double bonds (4). It exists in nature as an all-transform structure. It is the most abundant carotenoid in tomatoes and can also be found in apricots, melons, papayas, grapes, peaches, watermelons, and cranberries (4, 5). Studies have shown that lycopene possesses a variety of functions such as anti-oxidation (6, 7) anti-inflammation (8), immunomodulation (9), enhancement of gap junctional communication (10), induction of phase II enzymes (11), inhibition of cell proliferation (12), anti-cancer (13, 14), anti-angiogenesis (9, 15), and anti-metastasis (16–18). In anti-metastatic studies, lycopene has been found to inhibit the metastasis of human hepatoma SK-Hep-1 cells in athymic nude mice (17). Moreover, we recently reported that lycopene inhibits the metastasis of human liver adenocarcinoma SK-Hep-1 cells by downregulation of the NADPH oxidase 4 (NOX4) proteins (18). It is known that adjuvant is defined as a substance that helps and enhances the effect of a drug or treatment (19). To this topic, lycopene had been demonstrated to have synergism from the combination of oxaliplatin in a human ovarian cancer cell line (20), suggesting that the adjuvant property on the anticancer of lycopene. However, the adjuvant potential of lycopene against the metastasis of lung cancer cells remains unknown.

Sorafenib is a first-line drug used in the clinical treatment of advanced liver cancer and kidney cancer so as to improve the overall survival for the patients (21, 22). Side effects of sorafenib include loss of appetite, diarrhea, hair thinning, nausea or vomiting, hand-foot syndrome, or weight loss. Adjuvant for the sorafenib treatment may not only increase the anticancer efficacy, but also ease the side effects of sorafenib. Sorafenib is a multiple kinase inhibitor whose mechanism is to inhibit the Raf / MEK / ERK [i.e., the extracellular signal-regulated kinase (ERK) pathway] signaling to decrease the tumor growth and angiogenesis, and to induce the tumor cell apoptosis (23–25). In the colorectal cancer model of nude mice by subcutaneous injection of HuH7 cells, sorafenib (10 mg/kg) was combined with triptolide, and triptolide (0.21 mg/kg) can inhibit the ERK pathway and the AKT / mTOR pathway to reduce tumor growth (26). The anti-metastasis effect of sorafenib had been demonstrated by using the model of nude mice induced by *in situ* transplantation of the LM3-R cells to induce liver cancer (27); they found that sorafenib (30 mg/kg) given daily in combination with daily subcutaneous injection of 10 mg/kg zoledronic acid (ZA) or intraperitoneal injection of clodrolip 100 mg/kg twice a week for 35 days, and sorafenib combined with ZA or clodrolip is more effective in anti-metastasis than sorafenib alone (27). However, the lung anti-metastasis efficacy of sorafenib in combination with lycopene in mice remains unknown.

In this study, the effects of lycopene in combination with sorafenib to inhibit cancer metastasis in mice xenografted with the Lewis lung carcinoma (LLC) cells were investigated. We hypothesized that lycopene could have an additive effect with sorafenib to inhibit the lung metastasis of tumors in the C57BL/6 mice xenografted LLC cells, because lycopene and sorafenib may act at the same target of the mitogen-activated protein kinase (MAPK) signaling pathway. The MAPK pathway mainly can be divided into ERK, c-Jun N-terminal kinase (JNK), and p38 pathways (28). As described above, the ERK pathway should be one of the main targets that sorafenib can have as an anti-cancer property (23–26). In addition, a study showed that the expression of matrix metalloproteinase (MMP)-2 was enhanced by the reactive oxygen species (ROS) through ROS-MAPK axis i.e., via the activation of ERK, JNK, and p38 pathways by the increased ROS (29). In addition, it had been demonstrated that lycopene could increase the MMP-2, MMP-9, and NM23-H1 expression via the inhibition of the NADPH oxidase (NOX) 4 to inhibit the metastasis of SK-Hep-1 cells (18). NOXs make up the only known enzyme family with the sole function of producing ROS (30, 31). Because of the existence of the ROS-MAPK axis (29, 32, 33), the inhibition of NOX4 by lycopene, theoretically, will decrease the ROS in cells and then inactivate the three MAPK pathways. In addition, the anti-oxidative property of lycopene itself could also decrease the ROS level to inactivate the three MAPK pathways. Thus, we mechanistically hypothesized that the expression of the MMPs will be additively inhibited by the treatment of lycopene combined with sorafenib via the additive inhibition on the three MAPK pathways, and eventually have an additive effect on the anti-lung-metastasis of the tumor. Besides, the additive effects of lycopene and sorafenib on the other negative modulators of metastasis, including the tissue inhibitors of the matrix metalloproteinase (TIMP)-1 and TIMP-2, and NM23-H1, an anti-metastatic protein, were also evaluated.

## Materials and Methods

### Materials

All chemicals used were of analytical grade. Dulbecco's Modified Eagle Medium (DMEM), non-essential amino acid (NEAA), penicillin, sodium pyruvate, trypsin, fetal bovine serum (FBS) were purchased from GIBCO/BRL (Rockville, MD, USA). Lycopene (purity = 98%; Lot. CTN3163) was obtained from Wako (Osaka, Japan). Sorafenib was obtained from Bayer Corporation (West Haven, CT). Cremophor^®^ EL (polyoxyethylated castor oil), coin oil, -mercaptoethanol, bromophenol blue, glycerol, glycine, N, N, N', N'-tetramethylethylenediamine (TEMED), polyoxyethylenesorbitan monolaurate (TWEEN^®^ 20), sodium chloride, sodium dodecyl sulfate for electrophoresis (≥98.5%), Tris-HCl, Tris-base were purchased from Sigma Chemical company (St. Louis, MO, USA). Methanol (99.8%), 40% Bis-Acrylamide (29:1) solution obtained from Merck Chemical Company (Darmstadt, German). Immobilon™ PVDF (polyvinylidene fluoride transfer membranes), ECL chemiluminescent detection kit were obtained from Millipore Corporation (Beaford, MA, USA). Ammonium persulfate (APS) was obtained from GE Healthcare Bio-Sciences AB (Uppsala, Sweden). Mitogen-activated protein kinases (MAPKs) antibodies including p38, ERK1/2 and JNK1/2, and their phosphorylated protein were purchased from Cell Signaling Technology (Bevly, MA). The anti-NOX4 and tissue inhibitor of matrix metalloproteinase (TIMP)-1 antibody were purchased from Abcam (California, USA). Tissue inhibitor of matrix metalloproteinase (TIMP)-2 antibody, NM23-H1, secondary antibody anti-rabbit IgG, anti-mouse IgG were purchased from Genetex (California, USA).

### Cell Culture

Mouse lung cancer cell line [Lewis Lung carcinoma (LLC) cells] (BCRC NO. 60050) was purchased from the Biological Resources Conservation and Research Center of the Institute of Food Industry Development (Hsinchu, Taiwan). The culture medium used Dulbecco's Modified Eagle Medium (DMEM) medium supplemented with 10% (v/v) FBS, 0.37% (w/v) NaHCO3, penicillin (100 unit/mL), and cultured in CO2 incubator at 37°C and 5% CO2.

### Animals and Groups

Male mice of C57BL/6 strain (5 weeks old) were purchased from Lesco Biotechnology Co., Ltd. (Yilan, Taiwan). This animal study was approved by the Animal Management Committee of the National Chung Hsing University (Approval Number: 104-048). The mice were raised in the animal room with controlled room temperature 25 ± 2°C, humidity 65 ± 5%, and alternating 12-h-light/-dark cycles. After the mice were adapted for 1 week, the LLC cells (1 × 10^5^/100μl) were inoculated into the back of mice by subcutaneous injection. Primary tumor formation was visually observed approximately 9 days after the cancer cell injection. Following on, the mice were divided into five groups: (1) tumor control group; (2) Lycopene (5 mg/kg) alone; (3) Sorafenib (30 mg/kg) alone; (4) Sorafenib (30 mg/kg) + Lycopene (2 mg/kg); (5) Sorafenib (30 mg /kg) + lycopene (5 mg/kg). Mouse body weight and primary tumor area were measured twice weekly for a further 28 days and then sacrificed. During the rearing period, we used standard rodent chow and free access to water. The contents of the three major nutrients in the feed were 64% carbohydrate, 12% fat, and 24% protein, and each gram contained 3.18 kcal. The longest and shortest diameters of primary tumors were measured using calipers. The tumor area was calculated as π ÷ 4 × length × width.

### Administration of Lycopene and Sorafenib in Mice

Lycopene was dissolved in corn oil and dosed at 2 mg/kg and 5 mg/kg according to the mouse body weight. Lycopene was tube-fed twice a week. Sorafenib was dissolved in Cremophor^®^ EL/95 % ethanol (50:50) to prepare a four-fold stock solution every 3 days, which was stored at room temperature, diluted with sterile deionized water according to the mouse body weight to prepare a dose of 30 mg/kg. Sorafenib was administered by a feeding tube once daily. It is known that the corn oil and the Cremophor EL/95% ethanol have no anti-cancer activity and are frequently used as the vehicle of the agents for anti-cancer researches. Thus, we did not include both the vehicle controls into the study for the reduction of the used number of the tested animals.

### Organs Weighting and Lung Tumor Metastases Counting

After the mice were sacrificed, the dorsal primary tumor, lung, liver, spleen, and kidney were removed, weighed separately, and the number of tumor metastases to the lung was counted visually. Organs were stored at −80°C, or soaked in the 10% formalin, for subsequent experiments.

### Analysis of MMPs Activity by Zymography

The activities of MMP-2 and MMP-9 in the blood were measured using gelatin zymography according to a protocol with some minor modifications (18). The blood was collected by the orbital blood sampling procedures before the mice were sacrificed. The samples were stored in a centrifuge tube containing K3EDTA anticoagulant, centrifuged at 4°C, 3,000 rpm for 10 min, and the supernatant was collected and stored at −80°C for subsequent experiments. A proper volume of plasma was electrophoresed via 8% sodium dodecyl sulfate–polyacrylamide gel electrophoresis (SDS–PAGE) containing 0.1% gelatin. After electrophoresis, the gel was washed with 2.5% (v/v) Triton X-100 twice for 30 min and then incubated with reaction buffer (2 M Tris-HCl (pH 8.0), 1 M CaCl_2_, and 1% NaN_3_) for 12–15 h at 37°C. The gel was stained with Coomassie brilliant blue R-250 for 30 min and then destained in solutions containing 10% (v/v) acetic acid and 50% (v/v) methanol. The relative MMP-2 and MMP-9 activities were quantified using MatroxInspector version 2.1.

### Western Blotting

Protein expression of MMP-2, MMP-9, NOX4, MAPKs, TIMP-1, TIMP-2, and NM23-H1 were measured by Western blotting. 10 mg of the lung were added to 200L of tissue extract buffer, ground and centrifuged at 10,000 × g for 10 min at 4°C. The supernatant was collected and stored at −80°C. The proteins (40 μg) from the supernatant were resolved by SDS–PAGE and transferred onto a polyvinylidene fluoride (PVDF) membrane. After being blocked with Tris-buffered saline (TBS) buffer containing 5% non-fat milk, the membrane was washed three times with TBS buffer containing 0.1% (v/v) Tween 20 for 1 h and then incubated with the various primary antibodies at 4°C overnight. The membrane was incubated with a fluoresce-in-conjugated secondary antibody for 1 h and then detected with an ECL chemiluminescent detection kit (Amersham Co., Bucks, U.K.). The relative density of protein expression was quantified with AlphaEaseFC Analysis software.

### Histopathological Staining

The lung tissues were removed from the mice, immersed in 10 times the volume of 10% formalin buffer solution for fixation, and pulsated on a shaker overnight. The fixed tissue was rinsed with running water for 30 min, and then the tissue was dehydrated in 70%, 80%, 85%, 90%, and 95% alcohol for 1 hour. The tissue was then immersed 3 times in 100% alcohol for 1.5 h, then twice in xylene, and twice in liquid paraffin at 57°C for 2 hours. The tissue was embedded in liquid paraffin and cut into 2 μm sections with a tissue microtome. After staining with the hematoxylin and eosin, the tissue sections were observed with an optical microscope.

### Statistical Analysis

Data from more than three independent tests were analyzed using the analysis of variance (ANOVA), followed by the Least significant difference (LSD) mean comparisons using the SPSS v.14.0 software (SPSS, Inc., Chicago, USA). *P*-values <0.05 were considered statistically significant. Potential synergistic or additive effects of lycopene plus sorafenib were evaluated by comparing the total inhibition (or activation) obtained by the sum of the individual treatment's effects with the extent of inhibition (or activation) obtained by a combination of treatments, i.e., using observed percentage inhibition (or activation) and the formula: (the percent inhibition of lycopene + sorafenib) / [(the percent inhibition of lycopene) + (the percent inhibition of sorafenib)] (34). According to this formula, a value >1.0 is synergistic, a value of 0.5–1.0 is additive, while a value <0.5 is antagonistic (34). The same goes for the activation formula.

## Results

### Effects of Lycopene (Lyc) Alone, Sorafenib (SF) Alone, or Their Combination on the Body Weight and Organ Weight

**Figure 1:**
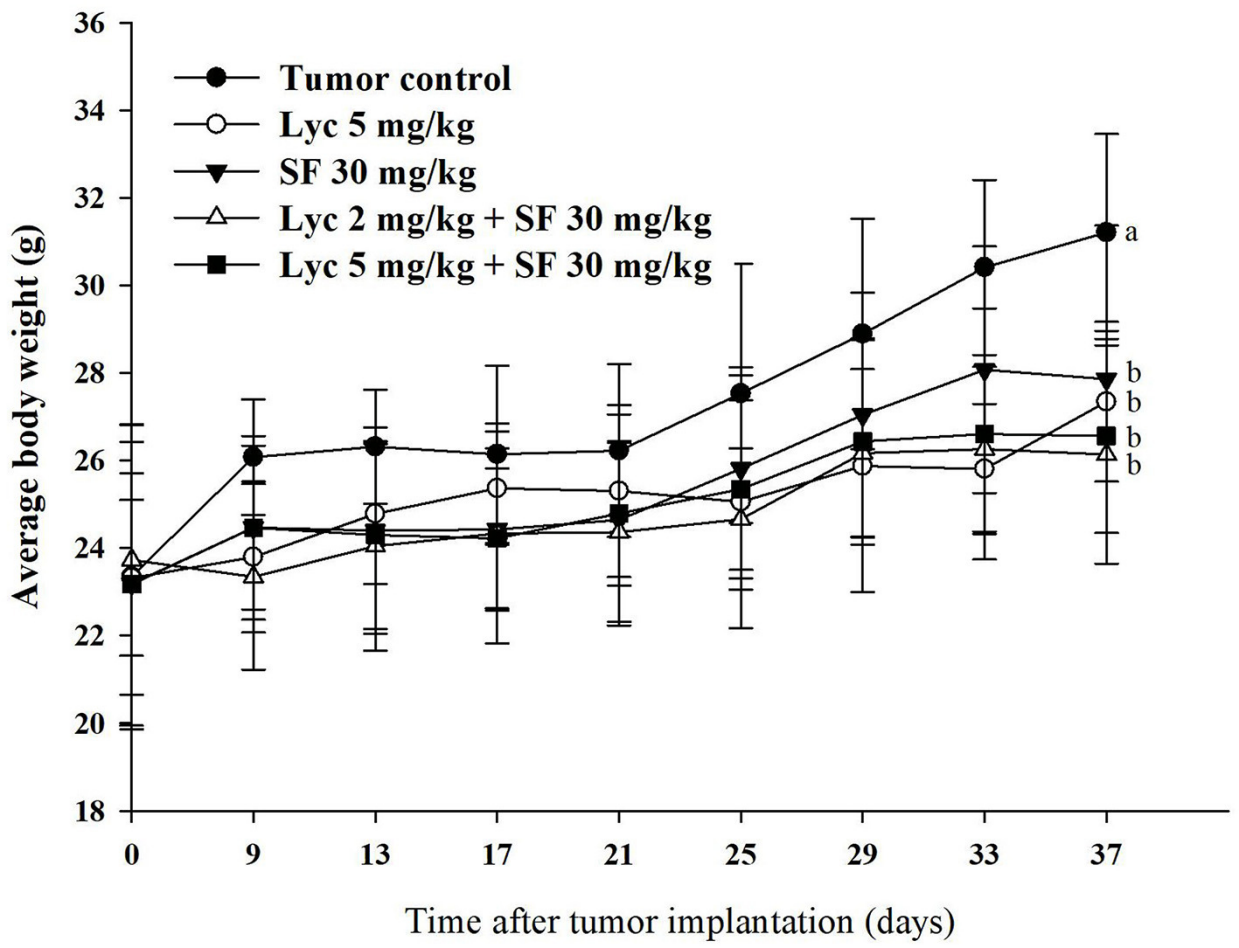
Effects of lycopene (Lyc) alone, sorafenib (SF) alone, or their combination on the body weight change in the C57BL/6 mice xenografted LLC cells during entire experimental period. LLC cells were injected subcutaneously into the right flank of the C57BL/6 mice. Nine days after the tumor cell injection, mice were orally supplemented with SF (30 mg/kg) daily, Lyc (5 mg/kg) twice per week or the combined treatment (mice were supplemented with SF (30 mg/kg) daily, and Lyc (2 and 5 mg/kg) twice per week) for 28 days. Data are means ± SD, *n* = 7–8; values not sharing an alphabetic letter are significantly different (*P* < 0.05).

As shown in Figure 1, the weight of each treatment group was significantly different from that of the control group at the end of the administrative day, (*P* < 0.05), but there was no significant difference between the treatment groups (*P* > 0.05). The organ weight, including the relative organ weight of liver, lung, spleen and kidney had no significant difference between the groups (Supplementary Table S1). The relative organ weight means that the organ weight was divided by the body weight of the mouse.

### Effects of Lycopene (Lyc) Alone, Sorafenib (SF) Alone, or Their Combination on the Lung Metastasis

**Figure 2:**
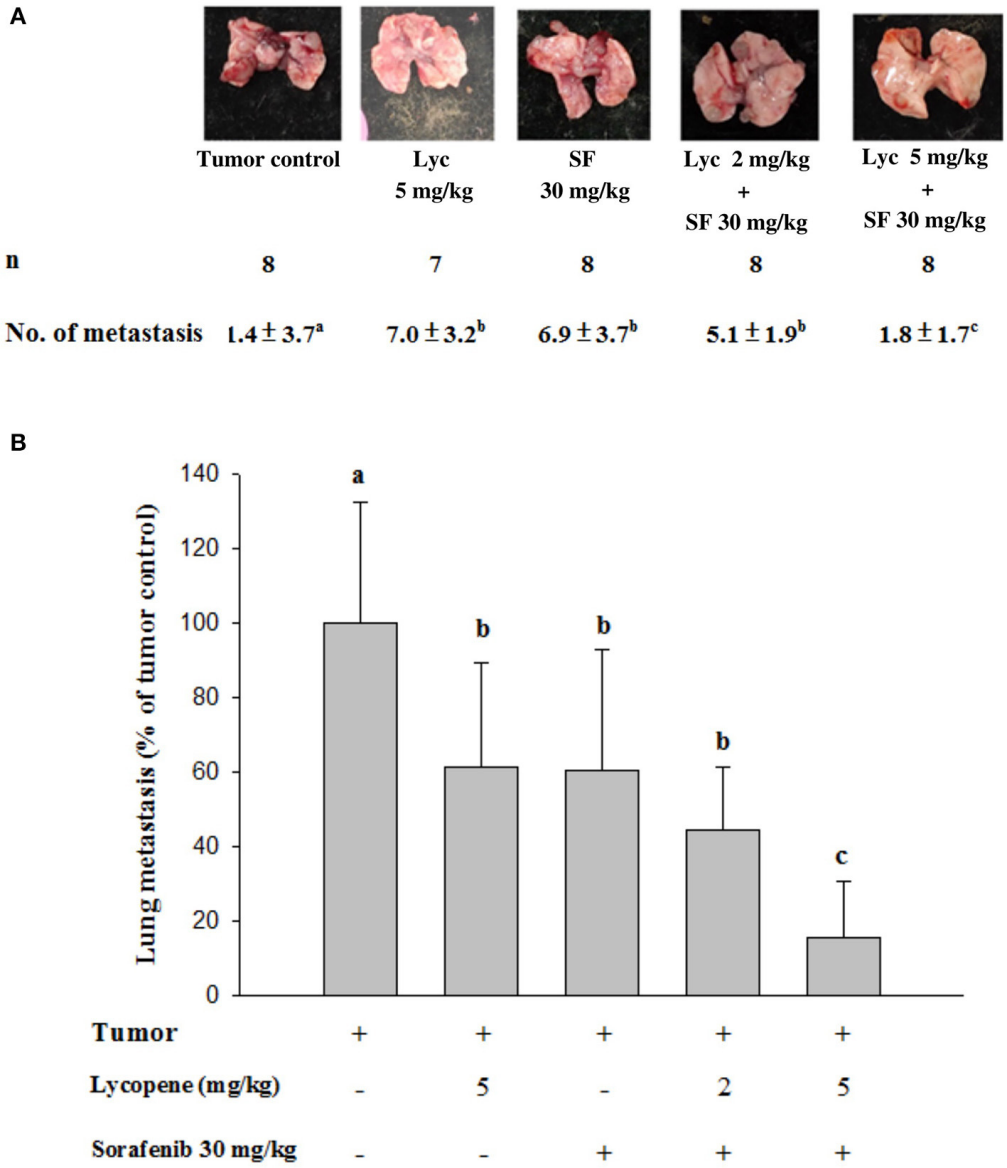
Effects of lycopene (Lyc) alone, sorafenib (SF) alone, or their combination on lung metastasis in the xenografted mice. LLC cells were injected subcutaneously into the right flank of the C57BL/6 mice. Nine days after the tumor cell injection, the mice were orally supplemented with SF (30 mg/kg) daily, Lyc (5 mg/kg) twice per week or the combined treatment (mice were supplemented with SF (30 mg/kg) daily, and Lyc (2 and 5 mg/kg) twice per week) for 28 days. **(A)** Macroscopic observation of lung metastasis. **(B)** The quantitative results of the lung metastasis. Data are means ± SD, *n* = 7–8; values not sharing an alphabetic letter are significantly different (*P* < 0.05).

Mice were sacrificed on day 37, and the tumor metastasis to the lungs of the mice was visually counted. The results showed that the administration of Lyc (5 mg/kg) and SF (30 mg/kg) alone significantly inhibited the number of tumors in the lungs as compared with the tumor control group, with the inhibition rates of 38.6 and 39.5%, respectively (*P* < 0.05, Table 1; Figure 2). Lyc (2 mg/kg) combined with SF (30 mg/kg) in the combined group could significantly reduce the number of lung tumor metastases as compared with the tumor control group, with an inhibition rate of 55.3% (*P* < 0.05, Table 1; Supplementary Figure S1). Compared with the tumor control group, the Lyc (5 mg/kg) combined with SF (30 mg/kg) group also significantly reduced tumor growth, with an inhibition rate of 84.2% (*P* < 0.05, Table 1; Figure 2). The fold of inhibition was 1.07, suggesting that a slight synergistic effect of lycopene in combination with sorafenib to inhibit the tumor number in lung as shown in Table 1.

**Table 1:** Inhibitory effects of lycopene (Lyc) alone, sorafenib (SF) alone, or their combination on the lung metastasis in the C57BL/6 mice xenografted LLC cells^1^.

**Groups**	***n***	**No. of metastasis (lung)**	**Inhibition (%)**	**Fold of inhibition (synergistic or additive effects)^2^**
Tumor control	8	11.4 ± 3.7^a^	-	-
Lyc 5 mg/kg	7	7.0 ± 3.2^b^	38.6	-
SF 30 mg/kg	8	6.9 ± 3.7^b^	39.5	-
Lyc 2 mg/kg + SF 30 mg/kg	8	5.1 ± 1.9^b^	55.3	-
Lyc 5 mg/kg + SF 30 mg/kg	8	1.8 ± 1.7^c^	84.2	1.07

1 *LLC cells (1 × 10^5^ cells/100L) were injected (s.c.) into C57BL/6 mice for 9 days, and the mice were orally administered with SF (30 mg/kg) daily, Lyc (5 mg/kg) twice per week, or the combined treatment (mice were supplemented with SF (30 mg/kg) daily, and Lyc (2 and 5 mg/kg) twice per week) for 28 days. After the mice were sacrificed, the tumor metastasis to the lungs of the mice was visually counted. Values are means ± SD from seven to eight mice. Values not sharing an alphabetic letter are significantly different (P < 0.05)*.

2 *The additive effect is calculated as (the percent inhibition of lycopene + sorafenib)/[(the percent inhibition of lycopene) + (the percent inhibition of sorafenib)]*.

### Effects of Lycopene (Lyc) Alone, Sorafenib (SF) Alone, or Their Combination on the Surface Area and Weight of Primary Tumor

**Figure 3:**
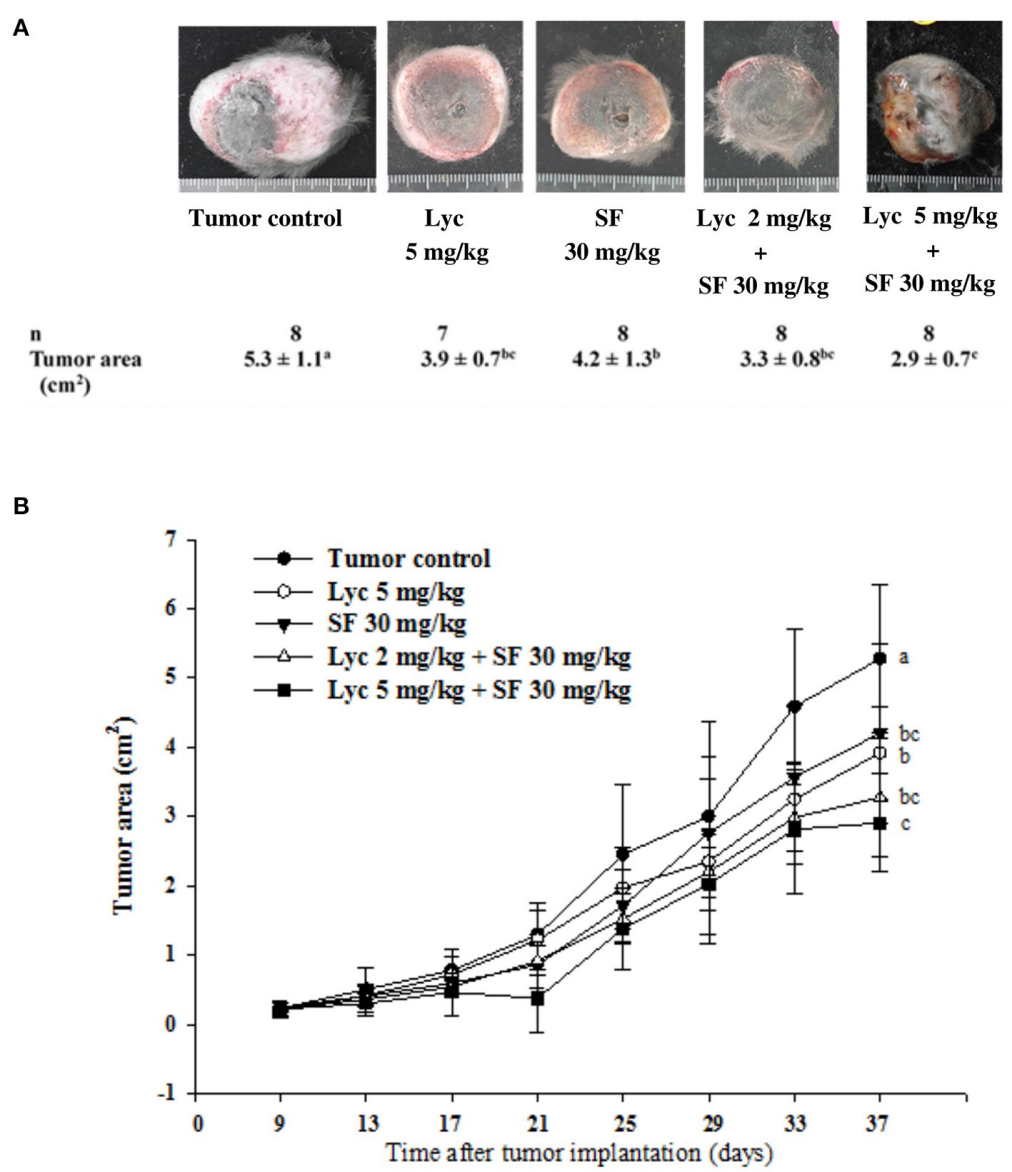
Effects of lycopene (Lyc) alone, sorafenib (SF) alone, or their combination on the primary tumor area in the xenografted mice during the entire experimental period. LLC cells were injected subcutaneously into the right flank of the C57BL/6 mice. Nine days after the tumor cell injection, the mice were orally supplemented with SF (30 mg/kg) daily, Lyc (5 mg/kg) twice per week or the combined treatment [mice were supplemented with SF (30 mg/kg) daily, and Lyc (2 and 5 mg/kg) twice per week] for 28 days. The formula for calculating the tumor area is: π ÷ 4 × length × width. **(A)** Images of primary tumor. **(B)** The quantitative results of the primary tumor area. Data are means ± SD, *n* = 7–8; values not sharing an alphabetic letter are significantly different (*P* < 0.05).

For the primary tumor area on day 37, the results showed that the administration of Lyc (5 mg/kg) or SF (30 mg/kg) alone significantly inhibited tumor growth as compared with the tumor control group, with the inhibition rates of 26.4 and 20.8%, respectively (*P* < 0.05, Table 2; Figure 3). Compared with the tumor control group, the inhibition rate of Lyc (2 and 5 mg/kg) combined with SF (30 mg/kg) was 37.7 and 45.3% (*P* < 0.05, Table 2; Figure 3). In the tumor weight, the results showed that the treatments could significantly reduce tumor weight as compared with the tumor control group no matter whether Lyc (5 mg/kg) and SF (30 mg/kg) were administered alone or in combination (*P* < 0.05, Table 2; Figure 3). The fold of inhibition was 0.96, suggesting that an additive effect of lycopene in combination with sorafenib to inhibit the area of primary tumor as shown in Table 2.

**Table 2:** Inhibitory effects of lycopene (Lyc) alone, sorafenib (SF) alone, or their combination on the primary tumor area and tumor weight in the C57BL/6 mice xenografted LLC cells^1^.

**Groups**	***n***	**Tumor weight (g)**	**Tumor area (cm^2^)**	**Tumor area inhibition (%)**	**Fold of tumor area inhibition (synergistic or additive effects)^2^**
Tumor control	8	9.2 ± 2.3^a^	5.3 ± 1.1^a^	-	-
Lyc 5 mg/kg	7	6.6 ± 1.6^b^	3.9 ± 0.7^bc^	26.4	-
SF 30 mg/kg	8	6.0 ± 2.3^b^	4.2 ± 1.3^b^	20.8	-
Lyc 2 mg/kg + SF 30 mg/kg	8	5.9 ± 1.0^b^	3.3 ± 0.8^bc^	37.7	-
Lyc 5 mg/kg + SF 30 mg/kg	8	4.8 ± 1.0^b^	2.9 ± 0.7^c^	45.3	0.96

1 *LLC cells (1 × 10^5^ cells/100L) were injected (s.c.) into the C57BL/6 mice for 9 days, and the mice were orally administered with SF (30 mg/kg) daily, Lyc (5 mg/kg) twice per week, or the combined treatment (mice were supplemented with SF (30 mg/kg) daily, and Lyc (5 mg/kg) twice per week) for 28 days. The results of the surface area and weight of the primary tumor on day 37 are shown. Values are means ± SD from seven to eight mice. Values not sharing an alphabetic letter are significantly different (P < 0.05)*.

2 *The additive effect is calculated as (the percent inhibition of tumor area of lycopene + sorafenib)/[(the percent inhibition of tumor area of lycopene) + (the percent inhibition of tumor area of sorafenib)]*.

### Effects of Lycopene (Lyc) Alone, Sorafenib (SF) Alone, or Their Combination on MMP-2 and MMP-9 Activity in Plasma

**Figure 4:**
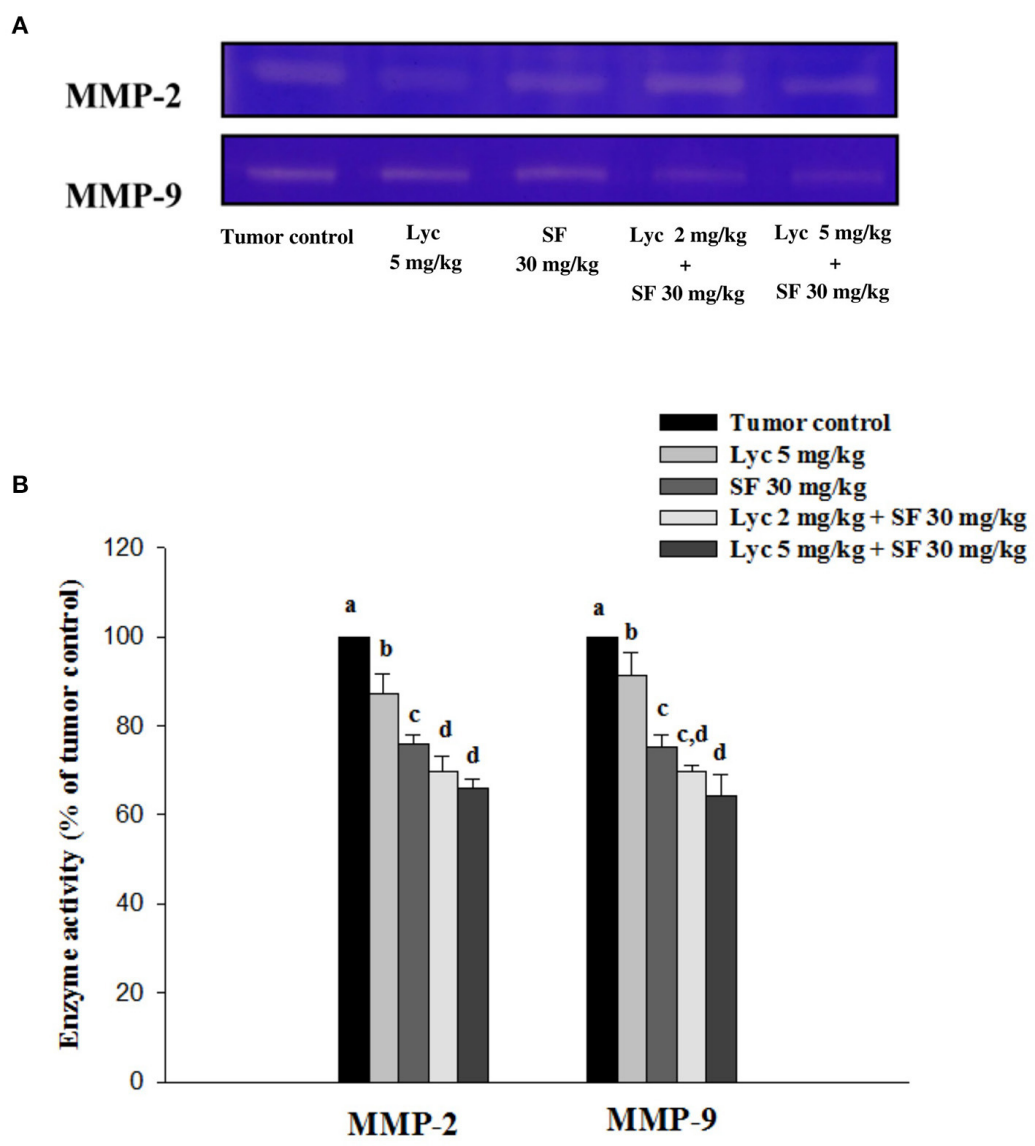
Effects of lycopene (Lyc) alone, sorafenib (SF) alone, or their combination on MMP-2 and MMP-9 activities in plasma of the C57BL/6 mice xenografted LLC cells. Nine days after the tumor cell injection, mice were orally supplemented with SF (30 mg/kg) daily, Lyc (5 mg/kg) twice per week or the combined treatment (mice were supplemented with SF (30 mg/kg) daily, and Lyc (2 and 5 mg/kg) twice per week) for 28 days. **(A)** Zymography analysis of MMP-2 and MMP-9 activities. **(B)** Quantitative results of zymography of MMP-2 and MMP-9 activities. Data are means ± SD, *n* = 7–8; values not sharing an alphabetic letter are significantly different (*P* < 0.05).

After the mice were sacrificed, the orbital blood of the mice was collected, and the enzymatic activities of MMP-2 and MMP-9 in the plasma were determined by zymography. The enzyme activities of MMP-2 and MMP-9 in the plasma of the tumor control group were set at 100%. The results showed that Lyc (5 mg/kg) alone could significantly inhibit MMP-2 and MMP-9 enzyme activities as compared to the tumor control group as the inhibition rates were 12.9% and 8.6%, respectively (*P* < 0.05, Figure 4; Supplementary Table S2); SF (30 mg/kg) given alone also significantly inhibited MMP-2 and MMP-9 enzyme activities as compared to the tumor control group as the inhibition rates were 24.1% and 24.9% (*P* < 0.05, Figure 4; Supplementary Table S2). The Lyc (2 mg/kg) combined with SF (30 mg/kg) group could significantly reduce the MMP-2 and MMP-9 enzyme activities as compared with the tumor control group, and the inhibition rates of both were about 30% (*P* < 0.05, Figure 4; Supplementary Table S2). Lyc (5 mg/kg) combined with SF (30 mg/kg) could significantly reduce the activity of MMP-2 and MMP-9 enzymes as compared with the tumor control group, with the inhibition rates of 34.2 and 35.6%, respectively (*P* < 0.05, Figure 4; Supplementary Table S2). The fold of inhibition in MMP-2 and MMP-9 were 0.92 and 1.07, respectively, suggesting an additive to a slightly synergistic effect of lycopene in combination with sorafenib on the inhibition of MMP-2 and MMP-9 activities are as shown in Supplementary Table S2.

### Effects of Lycopene (Lyc) Alone, Sorafenib (SF) Alone, or Their Combination on MMP-9 and MMP-2 Protein Expressions in Lung Tissues

**Figure 5:**
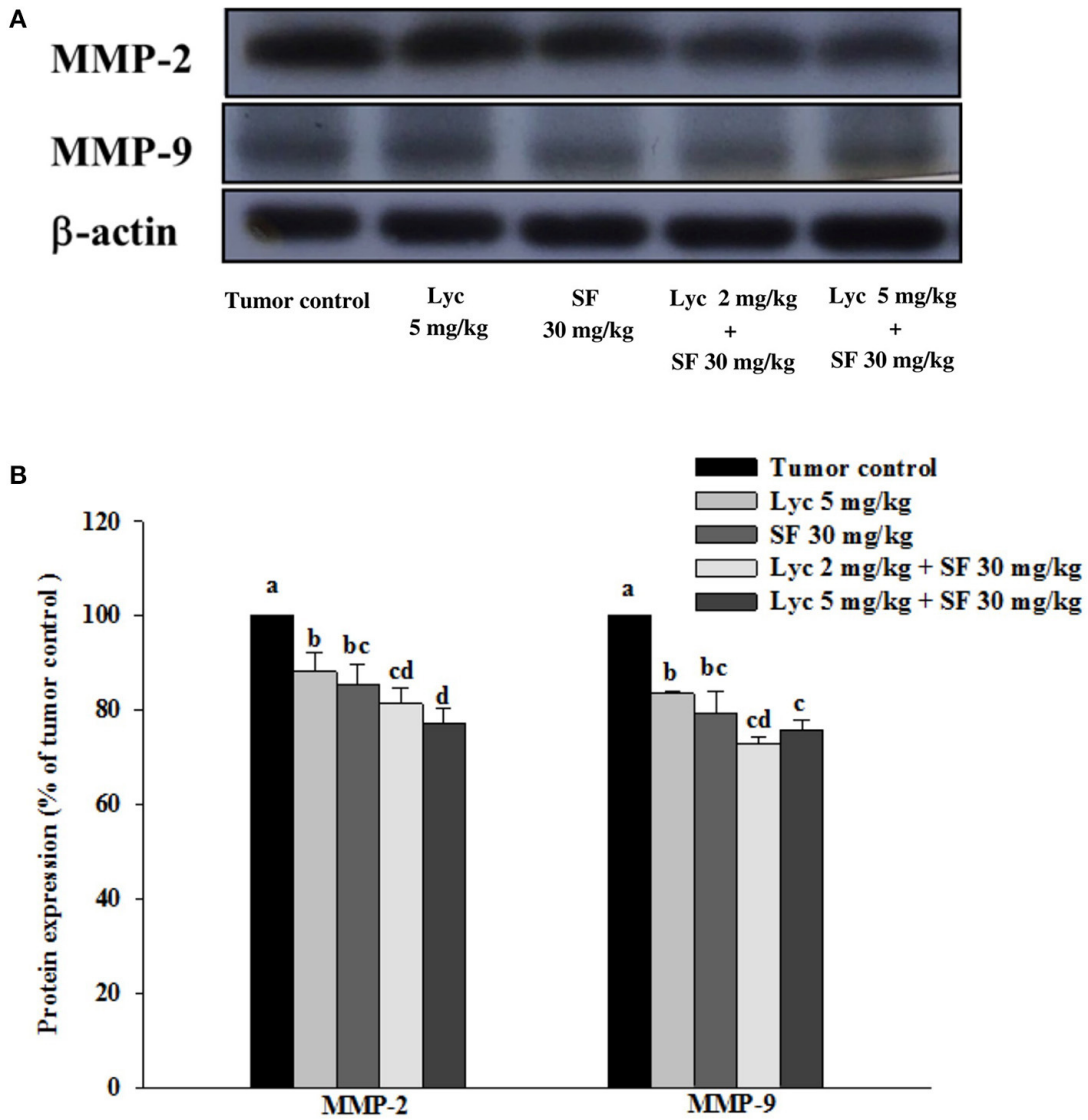
Effects of lycopene (Lyc) alone, sorafenib (SF) alone, or their combination on MMP-9 and MMP-2 protein expressions in lung tissues of the C57BL/6 mice xenografted LLC cells. Nine days after tumor cell injection, the mice were orally supplemented with SF (30 mg/kg) daily, Lyc (2 and 5 mg/kg) twice per week or the combined treatment (mice were supplemented with SF (30 mg/kg) daily, and Lyc (2 and 5 mg/kg) twice per week) for 28 days. **(A)** Western blot analysis of MMP-9, MMP-2 and-actin. **(B)** Quantitative results of the protein expressions of MMP-9 and MMP-2. Data are means ± SD, *n* = 7–8; values not sharing an alphabetic letter are significantly different (*P* < 0.05).

After the mice were sacrificed, the lung tissue was collected and homogenized, and the expression of MMP-2 and MMP-9 proteins in the lung tissue was analyzed by Western blot. The results showed that the group administered with Lyc (5 mg/kg) and SF (30 mg/kg) alone, compared with the tumor control group, significantly inhibited the protein expression of MMP-2 for 12.0 and 14.6% (*P* < 0.05, Figure 5; Supplementary Table S3). In the combined group, Lyc (2 mg/kg) combined with SF (30 mg/kg) significantly inhibited the protein expression of MMP-2 in the lungs, which was 18.7% (*P* < 0.05, Figure 5; Supplementary Table S3). The Lyc (5 mg/kg) combined with the SF (30 mg/kg) group also significantly inhibited the expression of MMP-2 protein in the lungs, with an inhibition rate of 22.9% (*P* < 0.05, Figure 5; Supplementary Table S3). Lycopene had similar results on the expression of MMP-9 (Figure 5; Supplementary Table S3). The fold of inhibition in MMP-2 and MMP-9 expressions were 0.86 and 0.65, respectively, suggesting that an additive effect of lycopene in combination with sorafenib on the inhibition of MMP-2 and MMP-9 expressions is as shown in Supplementary Table S3.

### Effects of Lycopene (Lyc) Alone, Sorafenib (SF) Alone, or Their Combination on the Phosphorylation of p38, ERK1/2 and JNK1/2 Proteins in Lung Tissues

**Figure 6:**
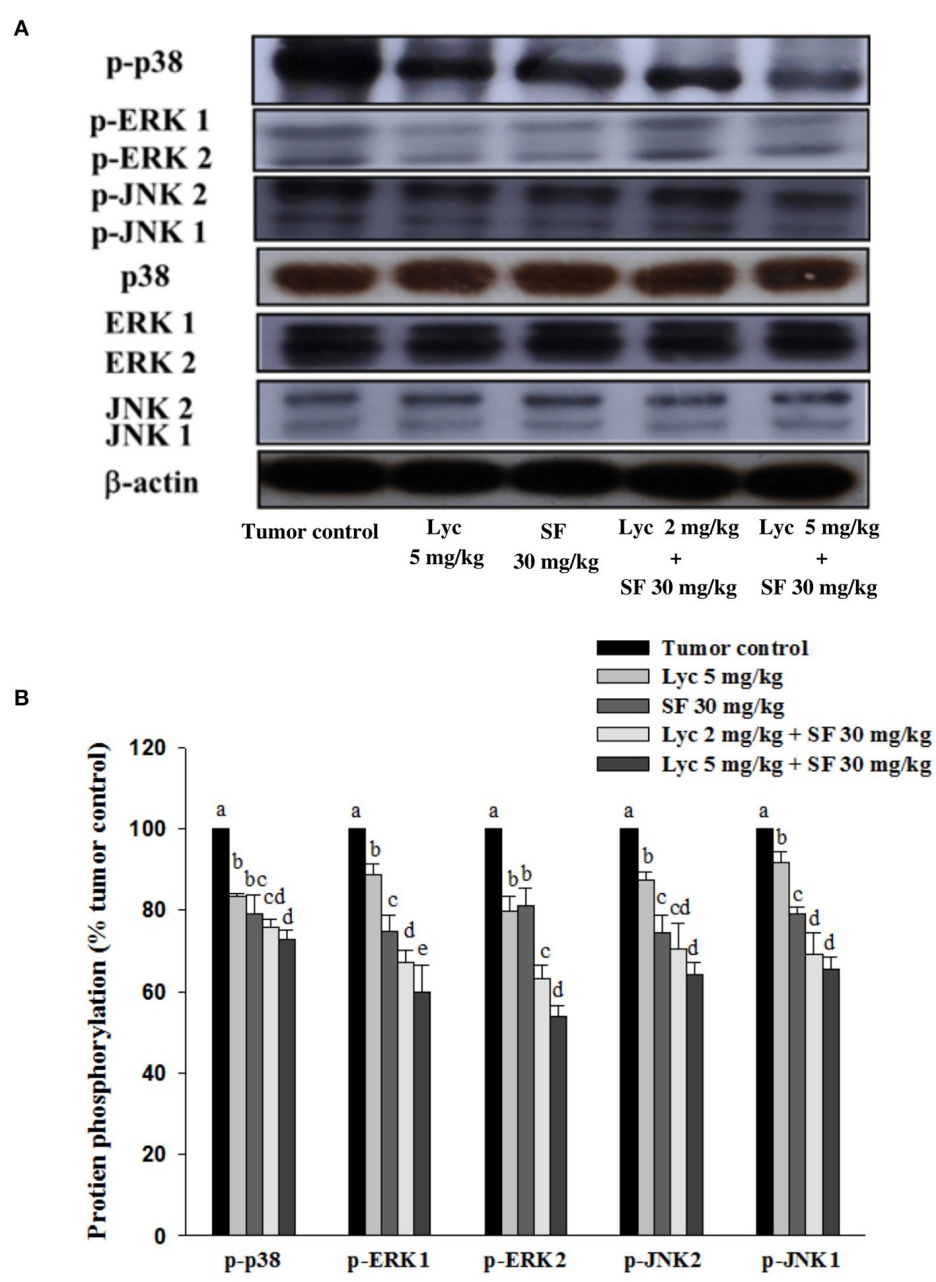
Effects of lycopene (Lyc) alone, sorafenib (SF) alone, or their combination on the phosphorylation of p38, ERK1/2 and JNK1/2 proteins in lung tissues of the C57BL/6 mice xenografted LLC cells. Nine days after tumor cell injection, the mice were orally supplemented with SF (30 mg/kg) daily, Lyc (5 mg/kg) twice per week or the combined treatment (mice were supplemented with SF (30 mg/kg) daily, and Lyc (2 and 5 mg/kg) twice per week) for 28 days. **(A)** Western blot analysis of p-p38, p38, p-ERK1/2, ERK1/2, p-JNK1/2, JNK1/2 and -actin. **(B)** Quantitative results of the protein phosphorylation of p38, ERK1/2, and JNK1/2. Data are means ± SD, *n* = 7–8; values not sharing an alphabetic letter are significantly different (*P* < 0.05).

After the mice were sacrificed, the lung tissue was collected and homogenized, and the phosphorylation of p38, ERK1/2 and JNK1/2 proteins in the lung tissue was analyzed by Western blot. The results showed that the group administered with Lyc (5 mg/kg) and SF (30 mg/kg) alone, compared with the tumor control group, significantly inhibited the phosphorylation of p38 for 16.7 and 20.9% (*P* < 0.05, Figure 6; Supplementary Table S4). In the combined group, Lyc (2 mg/kg) combined with the SF (30 mg/kg) significantly inhibited the phosphorylation of p38 in the lungs, which was 27.1% (*P* < 0.05, Figure 6; Supplementary Table S4). The Lyc (5 mg/kg) combined with the SF (30 mg/kg) group also significantly inhibited the phosphorylation of p38 in the lungs by 24.4% (*P* < 0.05, Figure 6; Supplementary Table S4). The fold of inhibition in the phosphorylation of p38 was 0.65, suggesting that an additive effect of lycopene in combination with sorafenib on the inhibition of p38 phosphorylation is as shown in Supplementary Table S4. Lycopene had similar results on the phosphorylation of ERK1/2 (Figure 6; Supplementary Table S4) and JNK1/2 (Figure 6; Supplementary Table S5), except the fold of inhibitions showed the additive with slightly synergistic effects.

### Effects of Lycopene (Lyc) Alone, Sorafenib (SF) Alone, or Their Combination on TIMP-1, TIMP-2 and NM23-H1 Protein Expressions in Lung Tissues

**Figure 7:**
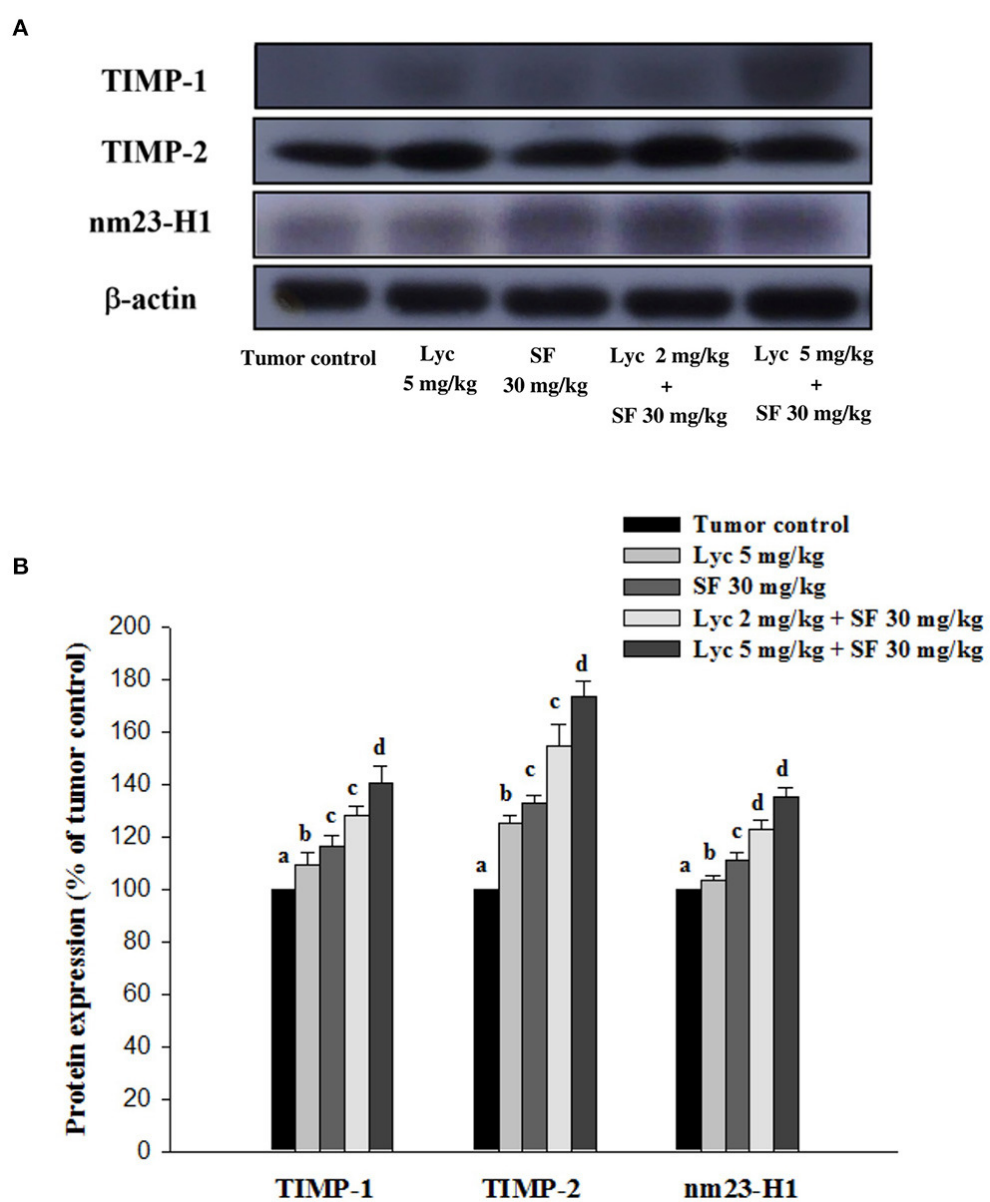
Effects of lycopene (Lyc) alone, sorafenib (SF) alone, or their combination on TIMP-1, TIMP-2 and nm23-H1 protein expression in liver tissues of the C57BL/6 mice xenografted LLC cells. Nine days after tumor cell injection, mice were orally supplemented with SF (30 mg/kg) daily, Lyc (5 mg/kg) twice per week or the combined treatment (mice were supplemented with SF (30 mg/kg) daily, and Lyc (2 and 5 mg/kg) twice per week) for 28 days. **(A)** Western blot analysis of TIMP-1, TIMP-2, nm23-H1 and -actin. **(B)** Quantitative results of the protein expressions of TIMP-1, TIMP-2 and nm23-H1. Data are means ± SD, *n* = 7–8; values not sharing an alphabetic letter are significantly different (*P* < 0.05).

After the mice were sacrificed, the lung tissue was collected and homogenized, and the expression of TIMP-1, TIMP-2, and NM23-H1 proteins in the lung tissue was analyzed by Western blot. The results showed that the group administered with Lyc (5 mg/kg) and SF (30 mg/kg) alone, compared with the tumor control group, significantly activated the protein expression of TIMP-1 for 9.6% and 16.7% (*P* < 0.05, Figure 7; Supplementary Table S6). In the combined group, Lyc (2 mg/kg) combined with SF (30 mg/kg) significantly activated the protein expression of TIMP-1 in the lungs, which was 28.2% (*P* < 0.05, Figure 7; Supplementary Table S6). The Lyc (5 mg/kg) combined with SF (30 mg/kg) group also significantly activated the expression of TIMP-1 protein in the lungs, with an activation rate of 40.8% (*P* < 0.05, Figure 7; Supplementary Table S6). The fold of activation in TIMP-1 expression was 1.55, suggesting that a synergistic effect of lycopene in combination with sorafenib on the activation of TIMP-1 expression is as shown in Supplementary Table S6. Lycopene had similar results on the expressions of TIMP-2 and NM23-H1 proteins (Figure 7; Supplementary Table S6).

### Effects of Lycopene (Lyc) Alone, Sorafenib (SF) Alone, or Their Combination on NOX4 Protein Expression in Lung Tissues

**Figure 8:**
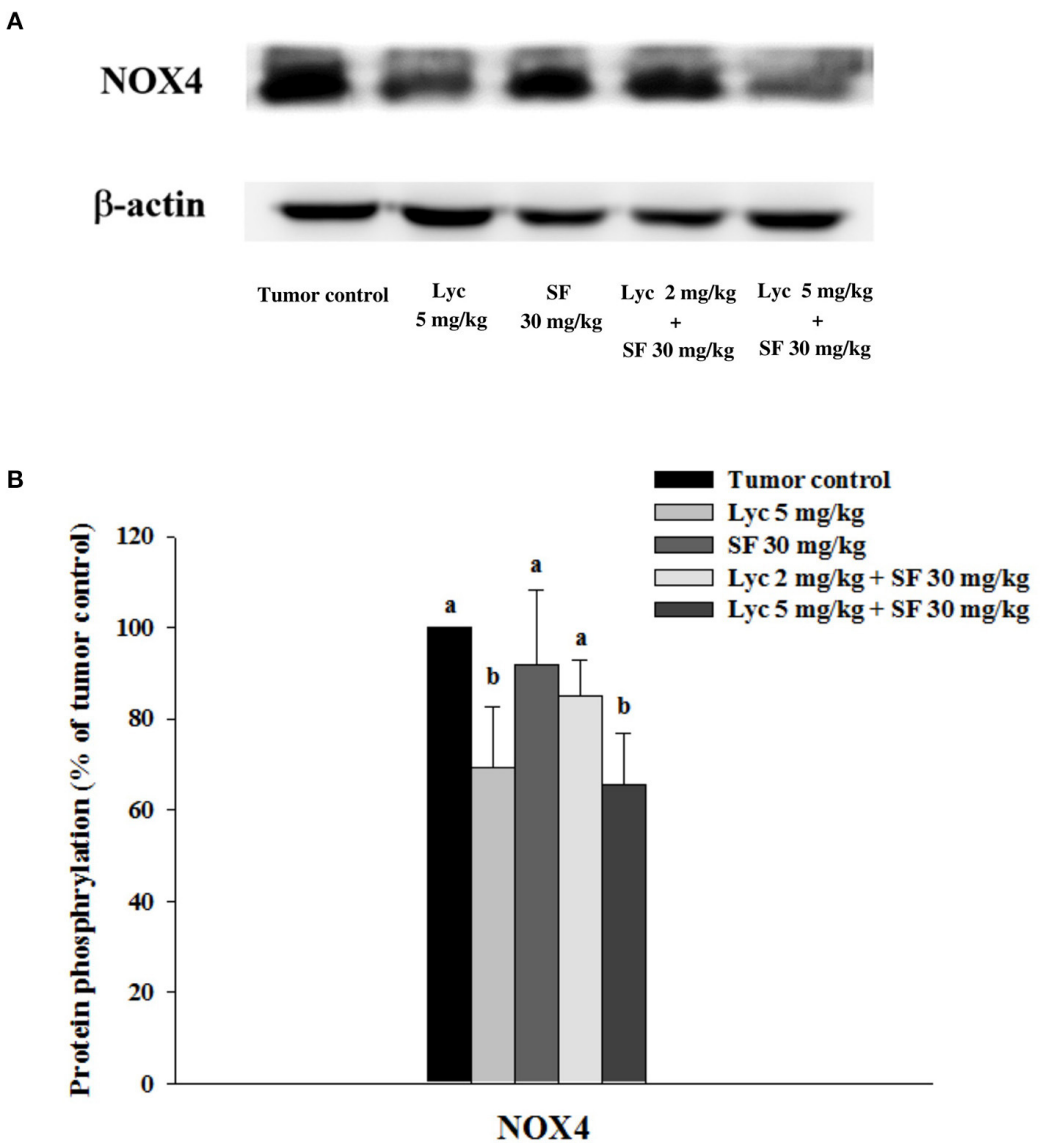
Effects of lycopene (Lyc) alone, sorafenib (SF) alone, or their combination on NOX4 protein expression in lung tissues of the C57BL/6 mice xenografted LLC cells. Nine days after the tumor cell injection, mice were orally supplemented with SF (30 mg/kg) daily, Lyc (5 mg/kg) twice per week or the combined treatment (mice were supplemented with SF (30 mg/kg) daily, and Lyc (2 and 5 mg/kg) twice per week) for 28 days. **(A)** Western blot analysis of NOX4 and -actin. **(B)** Quantitative results of the protein expression of NOX4. Data are means ± SD, *n* = 7–8; values not sharing an alphabetic letter are significantly different (*P* < 0.05).

After the mice were sacrificed, the lung tissue was collected and homogenized, and the expression of NOX4 protein in the lung tissue was analyzed by Western blot. The results showed that the group administered with Lyc (5 mg/kg) alone, compared with the tumor control group, significantly inhibited the protein expression of NOX4 for 30.6% (*P* < 0.05, Figure 8; Supplementary Table S7). The SF (30 mg/kg) alone had no significant inhibition on the expression of NOX4 (*P* > 0.05, Figure 8; Supplementary Table S7). The Lyc (5 mg/kg) combined with the SF (30 mg/kg) group showed a significant inhibition on the expression of NOX4 protein with an efficacy similar to the Lyc (5 mg/kg) alone group (*P* > 0.05) as compared to the Lyc (5 mg/kg) alone group (Figure 8; Supplementary Table S7).

### Effects of Lycopene (Lyc) Alone, Sorafenib (SF) Alone, or Their Combination on the Histopathological Analysis of Lung Tissues

As presented in Supplementary Figure S1 in the Supplementary Material, the results showed that the multiple, slight to moderate tumor cells were observed to metastasize to the lung via the blood or lymphatic vessels, forming tumor emboli (arrows in Supplementary Figure S1), and growing into the lung parenchyma in the groups of tumor control (A and B), Lyc alone (5 mg/kg) (C and D) and SF alone (30 mg/kg) (E and F) after the hematoxylin and eosin (H&E) staining of the lung sections. Alternatively, the multiple, minimal to slight, tumor cells were observed to metastasize to the lungs as the tumor emboli (arrows in Supplementary Figure S1), and growing into the lung parenchyma in the groups of Lyc (2 mg/kg) combined with SF (30 mg/kg) group (G and H), and the Lyc (5 mg/kg) combined with SF (30 mg/kg) group (I and J). While the tumor control group had multiple tumor foci, the Lyc showed a dose-dependent reduction with SF in the number of lung tumor foci.

## Discussion

A synergistic effect of phytochemicals such as lycopene and festin, in combination with anti-cancer drugs, has been found in the literature (19, 20). In this paper, we found that the combination of lycopene and sorafenib has a synergistic effect on the activation of TIMP-1, TIMP-2, and NM23-H1 protein expressions, and has an additive to the slightly synergistic effects on the inhibition of ERK-1, ERK-2, JNK-1, JNK-2, and p38 protein phosphorylation, and the expression of MMP-2 and MMP-9 proteins. In addition, lycopene can inhibit the lung metastasis of the xenografted tumor *in vivo*, and that the combination of lycopene and sorafenib has an additive effect on the lung metastasis. As shown, the numbers of metastasis tumor in the lung were addictively inhibited by the combination treatment. Moreover, the tumor area and weight *in situ* were also additively inhibited by the combination treatment. Taken together, lycopene combined with sorafenib can additively inhibit the primary tumor growth and the lung metastasis of the tumor in the xenografted mice. In addition, the results also support our hypothesis that the combined treatment of lycopene and sorafenib can simultaneously act at the three MAPK pathways, to a modulation of the MMP-2 and MMP-9 expressions, and then has an additive effect on the lung metastasis of the tumor in the xenografted mice. To the best of our knowledge, this paper is the first to report that lycopene and sorafenib can have an additive effect on the inhibition of primary tumor growth and the lung metastasis of tumors, suggesting that lycopene can be an adjuvant candidate for the treatment of cancer by sorafenib.

Cancer cells secrete proteolytic enzymes MMPs, which can degrade extracellular matrix and blood vessel walls, and have a high correlation with cancer metastasis (35, 36). The MMPs, MMP-2, and MMP-9 are the most related to tumor metastasis and invasion, because they have gelatinases activity and can degrade the extracellular matrix (37). The excessive expression of MMP-2 and MMP-9 is also highly correlated with lung cancer metastasis (38). In this study, the MMP-2 and MMP-9 activities and protein expressions showed an additive to the slightly synergistic effects for the combined treatment of lycopene and sorafenib. When the enzyme activity of MMPs are decreased, it can further reduce the degradation of extracellular matrix, thereby inhibiting the invasion of cancer cells and the metastasis of tumor to the lung.

The results in this study support our hypothesis that lycopene and sorafenib could act at the same target of the MAPK signaling pathways. The MAPK pathways have been demonstrated to be involved in many biological processes in cells including the metastasis of cancer cells (39–41). Among the MAPK pathways, the ERK pathway is activated by the epidermal growth factor receptors and Ras / Raf to promote cancer cell proliferation, survival, and metastasis (41). The JNK pathway can mainly regulate cell growth, differentiation and apoptosis and other physiological responses (42). The p38 pathway can directly phosphorylate downstream p53 and p73 to promote cell cycle arrest, apoptosis, cytokine secretion, etc., which in turn affects the cancer cell growth (43). Studies also showed that the MAPK signaling pathways are the upstream signals of MMP-2 (15, 29). In addition, several anti-metastasis agents had been demonstrated via the inhibition of the MAPK signaling pathways (44–46), suggesting that the MAPK pathways are an important target of anti-metastasis (41). Although it has been demonstrated that the ERK pathway is the main biological target of sorafenib, the results in this study demonstrate that all of the ERK, JNK, and p38 pathways can be the targets of sorafenib in the lung tissue of the xenografted mice. Importantly, the three MAPK pathways can be additively inhibited by the combination treatment with lycopene and sorafenib. Thus, the inhibition of MAPKs signaling should play a crucial role in the mechanism of the additive anti-metastasis of the combination treatment of lycopene and sorafenib through modulating the MMP-2 and MMP-9 activities.

In addition, other modulators of MMPs activity including NM23-H1 and TIMPs all showed with a synergistic effect by the combination treatment. NM23 is a family of metastasis suppressor genes, mainly isolated from the melanoma cells. At least eight human nm23 genes have been found, among which nm23-H1 and -H2 are the most widely studied, among which the nm23-H1 gene is highly related to the cancer metastasis (47). When NM23-H1 protein is overexpression, it can inhibit the invasion, migration and adhesion of cancer cells (47–49). TIMPs are endogenous inhibitors of MMPs. There are four genotypes of TIMPs found in humans: TIMP-1,−2,−3, and−4 (50). TIMP-1 and−2 are inhibitors of MMP-9 and−2, respectively, both of which are bonded in a 1:1 ratio by non-covalent bonds to inhibit the activity of MMPs (51). A study has also pointed out that TIMPs can down-regulate the expression of MMPs and inhibit the metastasis of cancer cells (52). Therefore, the balance between MMPs and TIMPs play an important role in cancer cell metastasis (52). However, the combination treatment showed a synergistic effect on the activation of NM23-H1, TIMP-1, and TIMP-2, which was not consistent with an additive effect of the combination treatment on lung metastases. The synergistic activation of these MMPs modulators only induces an additive reduction of the MMP-2 and MMP-9 activities, suggesting MMPs should be a critical control point for the ability of lung metastasis of the xenografted tumor. Nonetheless, the results in the present study support that NM23-H1, TIMP-1, and TIMP-2 are the upstream molecules before the MMP-2 and MMP-9 proteins by the lycopene and sorafenib alone or combinations.

**Figure 9:**
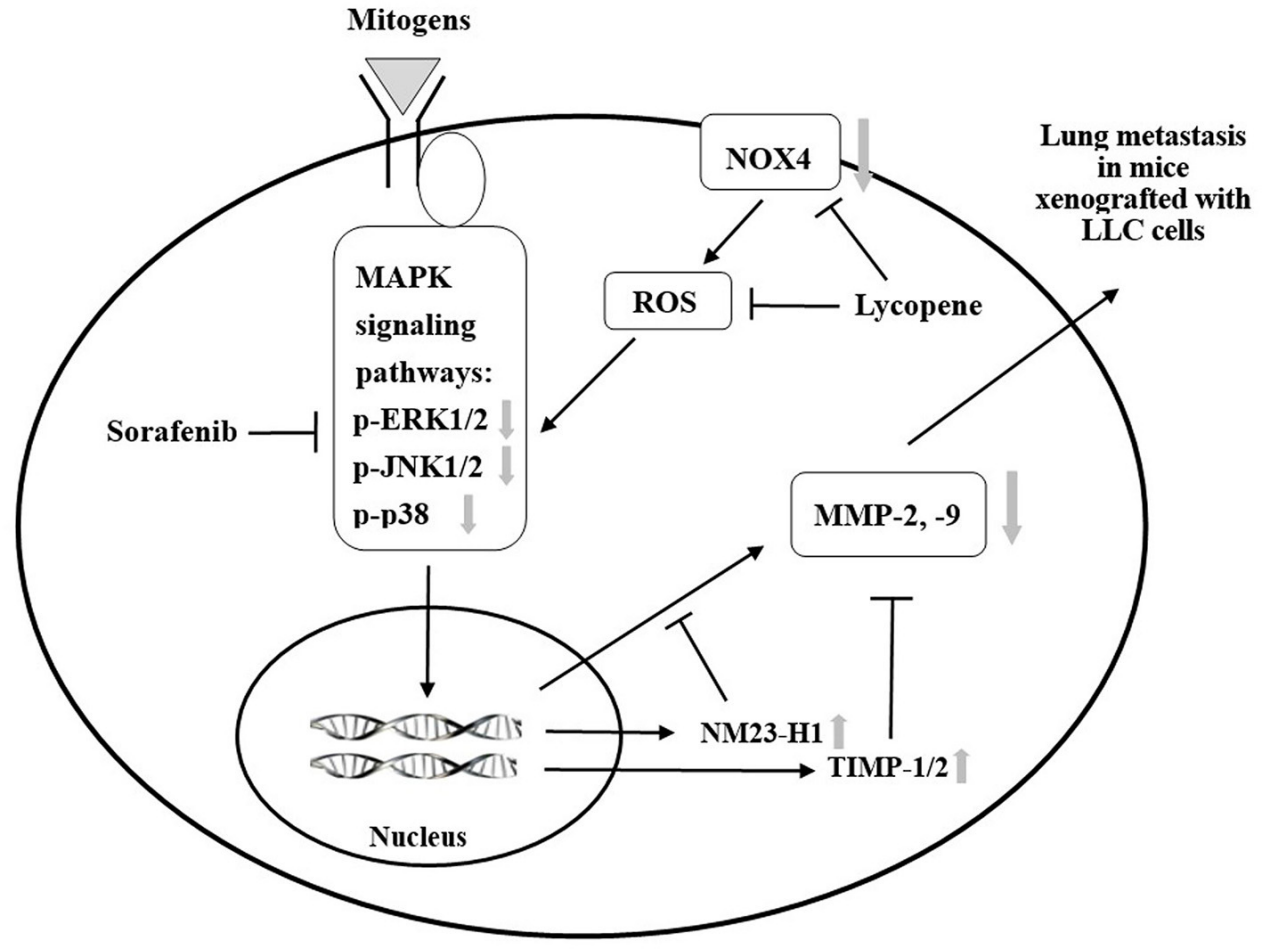
A proposed schematic diagram for the role of mitogen-activated protein kinase (MAPK) signaling pathways in the additive effect of lycopene and sorafenib in the C57BL/6 mice xenogrfied with the Lewis lung carcinoma (LLC) cells. 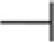: inhibition; 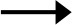: activation or expression. 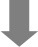: inhibition at the protein expression, phosphorylation or activity by the treatment of lycopene and sorafenib. 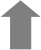: activation at the protein expression by the treatment of lycopene and sorafenib.

We have previously found that lycopene can inhibit the metastasis of human liver adenocarcinoma SK-Hep-1 cells by downregulation of the NOX4 protein expression *in vitro* (18). Herein, we confirm that lycopene could downregulate the NOX4 expression in the lung tissue of the mice xenografted with LLC cells *in vivo*. The results in this study support the existence of the NOX4 / ROS / MAPKs / MMPs pathway for lycopene. Besides, it should be noted that the anti-oxidative properties of lycopene itself may also play a role in its anti-metastatic action (6, 18). Based on these results and the literature review, we have proposed mechanisms related to the additively inhibit the lung metastasis of the xenografted tumor, as shown in Figure 9. Because the kinase-inhibiting property, the sorafenib can directly inhibit the MAPK pathways. In addition, lycopene inhibits the NOX4 activity and also inhibits the MAPK pathways. The additive inhibition of the MAPK pathways should decrease the MMP-2 and MMP-9 expressions through the activation of NM23-H1, TIMP-1, and TIMP-2. On the other hand, the additive inhibition of the MAPK pathways by lycopene and sorafenib may also contribute to the additive effects of these two compounds on primary tumor growth in the mice xenografted with LLC cells.

Previously, Huang et al., (17) had demonstrated that the lycopene supplementation resulted in the dose-dependent increases in lycopene concentrations in the lungs of the nude mice. In this study, since all of the effects of lycopene with sorafenib in the mice xenografted with LLC cells showed having a dose-dependent manner as well, suggesting that the anti-lung-metastatic activity, the observed MMPs activity and the effect on the MAPK signaling pathways etc. should be associated to the content of lycopene in the lungs. Furthermore, the dose of lycopene administered to mice in this article i.e., 5 mg/kg was further estimated to be converted into the amount required for intake for a human adult. We refer to the conversion formula proposed by Reagan-Shaw et al., (53): the human equivalent dosage (mg/kg) = animal dosage (mg/kg) × (animal Km value / hu-man Km value), where Km is the body weight (kg) ÷ body surface area (m2); human Km is approximately 37 and mouse Km is 3. The dosage of 5 mg/kg of lycopene was given to the mice twice a week, which was equivalent to 1.43 mg/kg of lycopene given to mice every day. Thus, the effective dosage of lycopene for a 60-kg healthy adults could be estimated as follows: 1.43 mg/kg/day × 60 kg × (3/37) = 6.96 mg/day of lycopene. Adults human need 6.69 mg/day to achieve an effective dosage of lycopene. Rao and Shen (54) pointed out that when the daily intake of lycopene in healthy adults reaches 57 mg, the concentration of lycopene in the blood may be sufficient to resist oxidative stress and various chronic diseases. A Canadian epidemiological study showed that the average daily intake of lycopene per woman is 6.14 ± 5.35 mg (*n* = 101) (55); another epidemiological study showed that the average daily dietary intake of lycopene in healthy women was 9.25 ± 6.43 mg (*n* = 632), and in breast cancer patients was 7.12 ± 6.73 mg (*n* = 122) (56). Thus, the effective dosage of lycopene to have an anti-metastasis efficacy i.e., 6.96 mg/day obtained by the results of this study is not difficult to achieve from a dietary intake.

## Conclusion

In summary, we demonstrate that lycopene can significantly inhibit the lung metastasis of tumors in the C57BL/6 mice xenografted with LLC cells *in vivo*, and we found that lycopene combined with the anticancer drug sorafenib can have an additive effect against the lung metastasis. The mechanisms of the additive anti-metastasis effect of lycopene and sorafenib are due to additively inhibit the MAPK signaling pathways, and decrease the MMP-2 and MMP-9 activities through the activation of the NM23-H1, TIMP-1, and TIMP-2 expressions. In addition, lycopene in combination with sorafenib also has an additive effect on the inhibition of tumor growth *in situ*. Based on these results, lycopene has a potential as an adjuvant for the cancer treatment of sorafenib.

## Data Availability Statement

The raw data supporting the conclusions of this article will be made available, without undue reservation, upon request to the corresponding author.

## Ethics Statement

The animal study was reviewed and approved by Animal Management Committee of the National Chung Hsing University.

## Author Contributions

N-CY and C-HC designed the study. Y-PC and IL participated in the experiments. Y-PC and N-CY wrote the draft. N-CY revised the manuscript. All authors approved the final manuscript.

## Funding

This work was supported by grants from the Ministry of Science and Technology of Taiwan (MOST 105-2320-B-040-017-MY3).

## Conflict of Interest

The authors declare that the research was conducted in the absence of any commercial or financial relationships that could be construed as a potential conflict of interest.

## Publisher's Note

All claims expressed in this article are solely those of the authors and do not necessarily represent those of their affiliated organizations, or those of the publisher, the editors and the reviewers. Any product that may be evaluated in this article, or claim that may be made by its manufacturer, is not guaranteed or endorsed by the publisher.
