# Supplementary material for: Lycopene in Combination With Sorafenib Additively Inhibits Tumor Metastasis in Mice Xenografted With Lewis Lung Carcinoma Cells
Source: Front Nutr. 2022 May 27;9:886988. doi: 10.3389/fnut.2022.886988 (PMC9197118; doi:10.3389/fnut.2022.886988)
Supplement: Supplementary file 1 [file Table_1.DOCX]

**Supplementary materials:**

**Table S1.** Effects of lycopene (Lyc) alone, sorafenib (SF) alone, or their combination on organ weight in the C57BL/6 mice xenografted LLC cells^1^

| Group | n | Organ weight (g) | | | | Relative organ weight (%)^2^ | | | |
| --- | --- | --- | --- | --- | --- | --- | --- | --- | --- |
|  |  | Liver | Lung | Spleen | Kidney | Liver | Lung | Spleen | Kidney |
| Tumor control | 8 | 1.31 ± 0.15 | 0.23 ± 0.08 | 0.26 ± 0.01 | 0.34 ± 0.04 | 4.6 ± 0.5 | 0.8 ± 0.2 | 0.92 ± 0.13 | 1.2 ± 0.2 |
| Lyc 5 mg/kg | 7 | 1.45 ± 0.17 | 0.28 ± 0.14 | 0.27 ± 0.04 | 0.34 ± 0.04 | 5.1 ± 0.5 | 1.0 ± 0.5 | 0.94 ± 0.12 | 1.2 ± 0.1 |
| SF 30 mg/kg | 8 | 1.21 ± 0.28 | 0.24 ± 0.06 | 0.26 ± 0.03 | 0.32 ± 0.03 | 4.4 ± 0.7 | 0.9 ± 0.3 | 0.96 ± 0.16 | 1.2 ± 0.1 |
| Lyc 2 mg/kg + SF 30 mg/kg | 8 | 1.17 ± 0.33 | 0.19 ± 0.05 | 0.27 ± 0.02 | 0.32 ± 0.07 | 4.7 ± 1.2 | 0.8 ± 0.3 | 1.09 ± 0.19 | 1.3 ± 0.2 |
| Lyc 5 mg/kg + SF 30 mg/kg | 8 | 1.28 ± 0.16 | 0.19 ± 0.06 | 0.27 ± 0.02 | 0.34 ± 0.04 | 4.6 ± 0.5 | 0.7 ± 0.2 | 0.98 ± 0.07 | 1.2 ± 0.1 |

^1^LLC cells (1 × 10^5^ cells/100 μL) were injected (s.c.) into C57BL/6 mice for nine days, and the mice were orally administered with SF (30 mg/kg) daily, Lyc (5 mg/kg) twice per week or the combined treatment (mice were supplemented with SF (30 mg/kg) daily, and Lyc (2 and 5 mg/kg) twice per week) for 28 days. Values are means ± SD from seven to eight mice. Values not sharing an alphabetic letter are significantly different (P < 0.05).

^2^Relative weights are calculated as % of individual weights.

**Table S2.** Effects of lycopene (Lyc) alone, sorafenib (SF) alone, or their combination on activities of MMP-2 and MMP-9 in plasma of the C57BL/6 mice xenografted LLC cells^1^

| Group | n | Enzyme activity (plasma) | | Inhibition (%) | | Fold of inhibition  (synergistic or additive effects)^2^ | |
| --- | --- | --- | --- | --- | --- | --- | --- |
|  |  | MMP-2 | MMP-9 | MMP-2 | MMP-9 | MMP-2 | MMP-9 |
| Tumor control | 8 | 100 ± 0^a^ | 100 ± 0^a^ | - | - | - | - |
| Lyc 5 mg/kg | 7 | 87.1 ± 4.5^b^ | 91.4 ± 5.0^b^ | 12.9 | 8.6 | - | - |
| SF 30 mg/kg | 8 | 75.9 ± 2.1^c^ | 75.1 ± 2.9^c^ | 24.1 | 24.9 | - | - |
| Lyc 2 mg/kg + SF 30 mg/kg | 8 | 69.9 ± 3.4^d^ | 69.7 ± 1.4^cd^ | 30.1 | 30.3 | - | - |
| Lyc 5 mg/kg + SF 30 mg/kg | 8 | 65.8 ± 2.3^d^ | 64.3 ± 4.8^d^ | 34.2 | 35.7 | 0.92 | 1.07 |

^1^ LLC cells (1 × 10^5^ cells/100 μL) were injected (s.c.) into C57BL/6 mice for nine days, and the mice were orally administered with SF (30 mg/kg) daily, Lyc (5 mg/kg) twice per week or the combined treatment (mice were supplemented with SF (30 mg/kg) daily, and Lyc (2 and 5 mg/kg) twice per week) for 28 days. The activities of MMP-2 and MMP-9 in plasma were determined. Values are means ± SD from seven to eight mice. Values not sharing an alphabetic letter are significantly different (P < 0.05).

^2^ The synergistic or additive effect is calculated as the fold of inhibition i.e., (the percent inhibition in lycopene + sorafenib combination group) / [(the percent inhibition in lycopene alone group) + (the percent inhibition in sorafenib alone group)].

**Table S3.** Effects of lycopene (Lyc) alone, sorafenib (SF) alone, or their combination on protein expressions of MMP-2 and MMP-9 in lung tissues of the C57BL/6 mice xenografted LLC cells^1^

| Group | n | Protein expression (%) | | Inhibition (%) | | Fold of inhibition  (synergistic or additive effects)^2^ | |
| --- | --- | --- | --- | --- | --- | --- | --- |
|  |  | MMP-2 | MMP-9 | MMP-2 | MMP-9 | MMP-2 | MMP-9 |
| Tumor control | 8 | 100 ± 0^a^ | 100 ± 0^a^ | - | - | - | - |
| Lyc 5 mg/kg | 7 | 88.0 ± 4.1^b^ | 83.3 ± 0.6^b^ | 12.0 | 16.7 | - | - |
| SF 30 mg/kg | 8 | 85.4 ± 4.1^bc^ | 79.1 ± 4.6^bc^ | 14.6 | 20.9 | - | - |
| Lyc 2 mg/kg + SF 30 mg/kg | 8 | 81.3 ± 3.4^cd^ | 72.9 ± 1.3^cd^ | 18.7 | 27.1 | - | - |
| Lyc 5 mg/kg + SF 30 mg/kg | 8 | 77.1 ± 3.1^d^ | 75.6 ± 2.1^c^ | 22.9 | 24.4 | 0.86 | 0.65 |

^1^LLC cells (1 × 10^5^ cells/100 μL) were injected (s.c.) into the C57BL/6 mice for nine days, and the mice were orally administered with SF (30 mg/kg) daily, Lyc (5 mg/kg) twice per week or the combined treatment (mice were supplemented with SF (30 mg/kg) daily, and Lyc (2 and 5 mg/kg) twice per week) for 28 days. The protein expressions of MMP-2 and MMP-9 in the lung tissues were determined. Values are means ± SD from seven to eight mice. Values not sharing an alphabetic letter are significantly different (P < 0.05).

^2^ The synergistic or additive effect is calculated as the fold of inhibition i.e., (the percent inhibition in lycopene + sorafenib combination group) / [(the percent inhibition in lycopene alone group) + (the percent inhibition in sorafenib alone group)].

**Table S4.** Effects of lycopene (Lyc) alone, sorafenib (SF) alone, or their combination on the phosphorylation of p38 and ERK1/2 proteins in lung tissues of the C57BL/6 mice xenografted LLC cells^1^

| Group | n | Protein expression (%) | | | Inhibition (%) | | | Fold of inhibition  (synergistic or additive effects)^2^ | | |
| --- | --- | --- | --- | --- | --- | --- | --- | --- | --- | --- |
|  |  | p-p38 | p-ERK1 | p-ERK2 | p-p38 | p-ERK1 | p-ERK2 | p-p38 | p-ERK1 | p-ERK2 |
| Tumor control | 8 | 100 ± 0^a^ | 100 ± 0^a^ | 100 ± 0^a^ | - | - | - | - | - | - |
| Lyc 5 mg/kg | 7 | 83.3 ± 0.6^b^ | 88.6 ± 2.9^b^ | 79.9 ± 3.6^b^ | 16.7 | 11.5 | 20.1 | - | - | - |
| SF 30 mg/kg | 8 | 79.1 ± 4.6^bc^ | 74.7 ± 4.0^c^ | 81.0 ± 4.2^b^ | 20.9 | 25.3 | 19.0 | - | - | - |
| Lyc 2 mg/kg + SF 30 mg/kg | 8 | 75.6 ± 2.1^cd^ | 67.1 ± 3.2^d^ | 63.3 ± 3.3^c^ | 27.1 | 32.9 | 36.7 | - | - | - |
| Lyc 5 mg/kg + SF 30 mg/kg | 8 | 72.9 ± 2.1^d^ | 60.0 ± 6.4^e^ | 54.0 ± 2.4^d^ | 24.4 | 40.0 | 46.0 | 0.65 | 1.09 | 1.18 |

^1^LLC cells (1 × 10^5^ cells/100 μL) were injected (s.c.) into the C57BL/6 mice for nine days, and the mice were orally administered with SF (30 mg/kg) daily, Lyc (5 mg/kg) twice per week or the combined treatment (mice were supplemented with SF (30 mg/kg) daily, and Lyc (2 and 5 mg/kg) twice per week) for 28 days. Values are means ± SD from seven to eight mice. Values not sharing an alphabetic letter are significantly different (*P* < 0.05).

^2^The synergistic or additive effect is calculated as the fold of inhibition i.e., (the percent inhibition in lycopene + sorafenib combination group) / [(the percent inhibition in lycopene alone group) + (the percent inhibition in sorafenib alone group)].

**Table S5.** Effects of lycopene (Lyc) alone, sorafenib (SF) alone, or their combination on the phosphorylation of JNK1 and JNK2 in lung tissues of the C57BL/6 mice xenografted LLC cells^1^

| Group | n | Protein expression (%) | | Inhibition (%) | | Fold of inhibition  (synergistic or additive effects)^2^ | |
| --- | --- | --- | --- | --- | --- | --- | --- |
|  |  | p-JNK2 | p-JNK1 | p-JNK2 | p-JNK1 | p-JNK2 | p-JNK1 |
| Tumor control | 8 | 100 ± 0^a^ | 100 ± 0^a^ | - | - | - | - |
| Lyc 5 mg/kg | 7 | 87.3 ± 2.0^b^ | 91.8 ± 2.6^b^ | 12.7 | 8.2 | - | - |
| SF 30 mg/kg | 8 | 74.6 ± 4.2^c^ | 79.0 ± 1.6^c^ | 25.4 | 21.0 | - | - |
| Lyc 2 mg/kg + SF 30 mg/kg | 8 | 70.5 ± 6.2^cd^ | 69.0 ± 5.6^d^ | 29.5 | 31.0 | - | - |
| Lyc 5 mg/kg + SF 30 mg/kg | 8 | 64.1 ± 3.2^d^ | 65.4 ± 3.2^d^ | 35.9 | 34.6 | 0.94 | 1.18 |

^1^LLC cells (1 × 10^5^ cells/100 μL) were injected (s.c.) into the C57BL/6 mice for nine days, and the mice were orally administered with SF (30 mg/kg) daily, Lyc (5 mg/kg) twice per week or the combined treatment (mice were supplemented with SF (30 mg/kg) daily, and Lyc (2 and 5 mg/kg) twice per week) for 28 days. Values are means ± SD from seven to eight mice. Values not sharing an alphabetic letter are significantly different (P < 0.05).

^2^The synergistic or additive effect is calculated as the fold of inhibition i.e., (the percent inhibition in lycopene + sorafenib combination group) / [(the percent inhibition in lycopene alone group) + (the percent inhibition in sorafenib alone group)].

**Table S6.** Effects of lycopene (Lyc) alone, sorafenib (SF) alone, or their combination on protein expression of TIMP-1, TIMP-2 and NM23-H1 in lung tissues of the C57BL/6 mice xenografted LLC cells^1^

| Group | n | Protein expression (%) | | | | Activation (%) | | | | Fold of activation  (synergistic or additive effects)^2^ | | | |
| --- | --- | --- | --- | --- | --- | --- | --- | --- | --- | --- | --- | --- | --- |
|  |  | TIMP-1 | TIMP-2 | NM23-H1 | TIMP-1 | | TIMP-2 | NM23-H1 | TIMP-1 | | TIMP-2 | NM23-H1 |  |
| Tumor control | 8 | 100 ± 0^a^ | 100 ± 0^a^ | 100 ± 0^a^ | - | | - | - | - | | - | - |  |
| Lyc 5 mg/kg | 7 | 109.6 ± 4.2^b^ | 125.0 ± 3.3^b^ | 103.3 ± 1.7^b^ | 9.6 | | 25.0 | 3.3 | - | | - | - |  |
| SF 30 mg/kg | 8 | 116.7 ± 3.7^c^ | 132.6 ± 3.4^c^ | 111.3 ± 2.6^c^ | 16.7 | | 32.6 | 11.3 | - | | - | - |  |
| Lyc 2 mg/kg + SF 30 mg/kg | 8 | 128.2 ± 3.6^c^ | 154.6 ± 8.5^c^ | 122.6 ± 4.0^d^ | 28.2 | | 54.6 | 22.6 | - | | - | - |  |
| Lyc 5 mg/kg + SF 30 mg/kg | 8 | 140.8 ± 6.2^d^ | 173.7 ± 5.5^d^ | 135.1 ± 3.9^d^ | 40.8 | | 73.7 | 35.1 | 1.55 | | 1.28 | 2.40 |  |

^1^LLC cells (1 × 10^5^ cells/100 μL) were injected (s.c.) into the C57BL/6 mice for nine days, and the mice were orally administered with SF (30 mg/kg) daily, Lyc (5 mg/kg) twice per week or the combined treatment (mice were supplemented with SF (30 mg/kg) daily, and Lyc (2 and 5 mg/kg) twice per week) for 28 days. The protein expressions of TIMP-1 and TIMP-2 in the lung tissues were determined. Values are means ± SD from seven to eight mice. Values not sharing an alphabetic letter are significantly different (P < 0.05).

^2^The synergistic or additive effect is calculated as the fold of activation i.e., (the percent activation in lycopene + sorafenib combination group) / [(the percent activation in lycopene alone group) + (the percent activation in sorafenib alone group)].

**Table S7.** Effects of lycopene (Lyc) alone, sorafenib (SF) alone, or their combination on protein expression of NOX4 in lung tissues of the C57BL/6 mice xenografted LLC cells^1^

| Group | n | Protein expression (%) | Inhibition (%) |
| --- | --- | --- | --- |
|  |  | NOX4 | NOX4 |
| Tumor control | 8 | 100 ± 0^a^ | - |
| Lyc 5 mg/kg | 7 | 69.4 ± 13.3^b^ | 30.6 |
| SF 30 mg/kg | 8 | 91.7 ± 16.5^a^ | 8.3 |
| Lyc 2 mg/kg + SF 30 mg/kg | 8 | 84.9 ± 7.9^a^ | 15.1 |
| Lyc 5 mg/kg + SF 30 mg/kg | 8 | 65.7 ± 11.2^b^ | 34.3 |

^1^LLC cells (1 × 10^5^ cells/100 μL) were injected (s.c.) into the C57BL/6 mice for nine days, and the mice were orally administered with SF (30 mg/kg) daily, Lyc (5 mg/kg) twice per week or the combined treatment (mice were supplemented with SF (30 mg/kg) daily, and Lyc (2 and 5 mg/kg) twice per week) for 28 days. Values are means ± SD from seven to eight mice. Values not sharing an alphabetic letter are significantly different (P < 0.05).


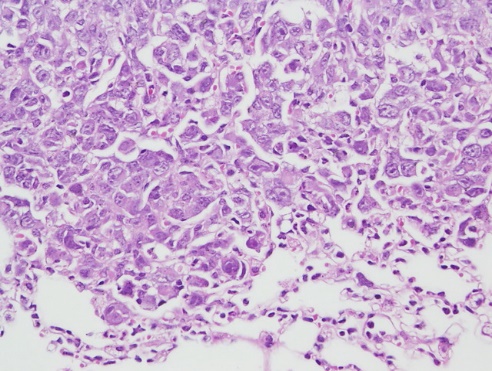

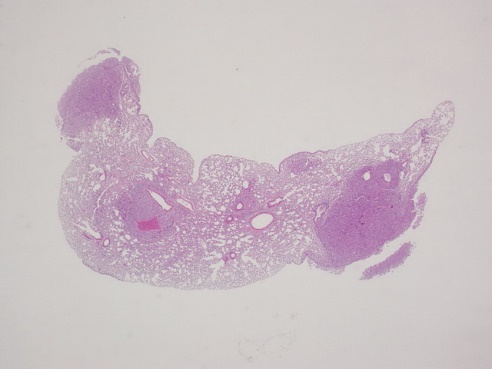

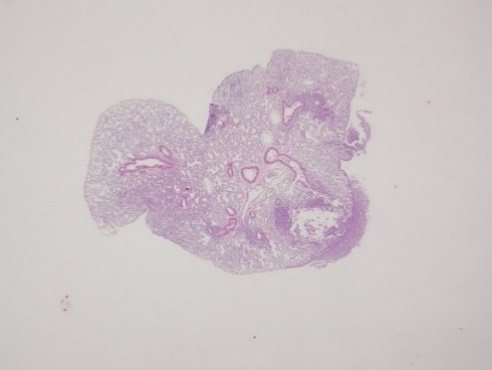

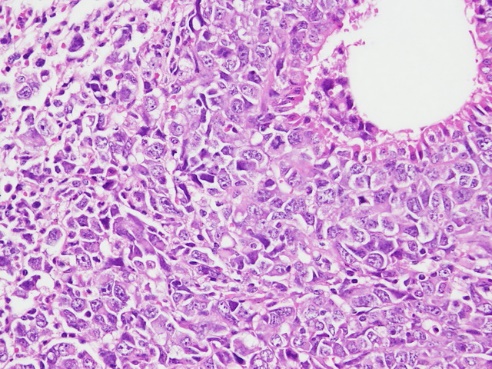

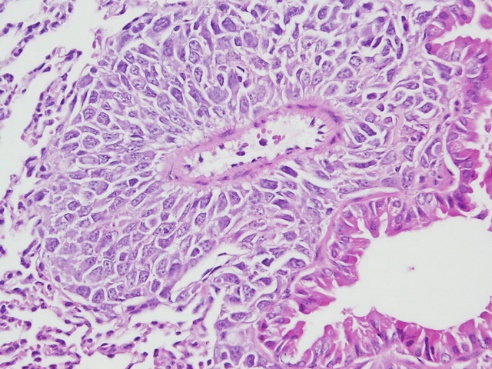

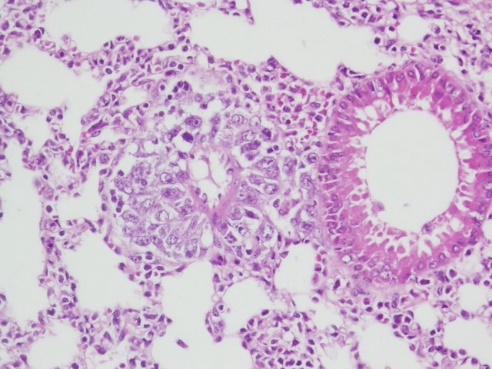

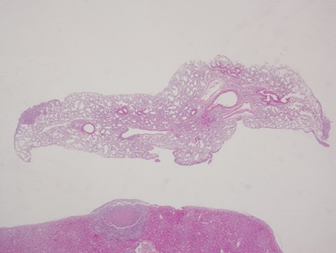

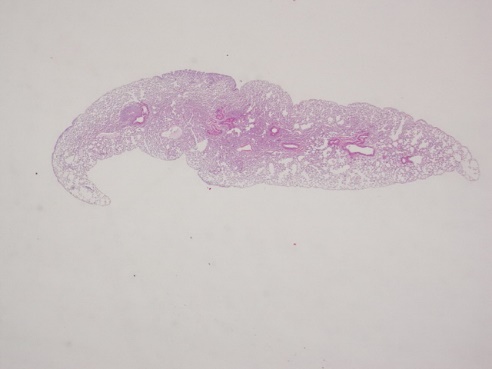

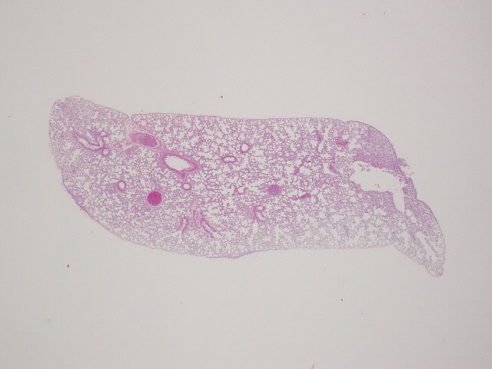

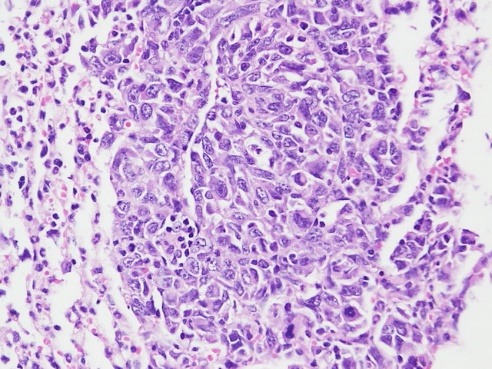


E

A

B

D

C

F

G

H

I

J

**Figure S1**. The Histopathological findings of the lungs of the C57BL/6 mice xenografted with the Lewis lung carcinoma (LLC) cells. The tumor control group (A and B), Lyc 5 mg/kg (C and D), SF 30 mg/kg (E and F), Lyc 2 mg/kg + SF 30 mg/kg (G and H), and Lyc 5 mg/kg + SF 30 mg/kg (I and J). H&E stain. 20x (right), 400x (left).
